# Supplementary material for: Molecular Profiling Reveals Characteristic and Decisive Signatures in Patients after Allogeneic Stem Cell Transplantation Suffering from Invasive Pulmonary Aspergillosis
Source: J Fungi (Basel). 2022 Feb 10;8(2):171. doi: 10.3390/jof8020171 (PMC8880021; doi:10.3390/jof8020171)

Supplementary material

Supplementary Table S1: Clinical information of alloSCT patients investigated in the study. Patient samples were collected at the University Hospital of Würzburg (Würzburg, Germany) and Public Health Wales Microbiology Cardiff (Cardiff, United Kingdom).

**Patient ID**	**Sex**	**Age**	**Origin**	**Status**	**Underlying disease**	**Cohort**
Ca1	M	56	UK	Probable IPA	AML	Cardiff I
Ca2	M	46	UK	Probable IPA	Myelofibrosis	Cardiff I
Ca3	M	52	UK	Probable IPA	AML	Cardiff I
Ca4	M	67	UK	Probable IPA	AML	Cardiff I
Ca5	F	24	UK	Probable IPA	CIVD	Cardiff I
Ca6	F	65	UK	Probable IPA	AML	Cardiff I
Ca7	MF	52	UK	Control	AML/MDS	Cardiff I
Ca8	F	71	UK	Control	AML	Cardiff I
Ca9	M	64	UK	Control	MS	Cardiff I
Ca10	F	64	UK	Control	MDS	Cardiff I
Ca11	M	18	UK	Control	ALL	Cardiff I
Ca12	M	69	UK	Control	AML	Cardiff I
Ca13	F	24	UK	Possible IPA	CGD	Cardiff I
Ca14	M	55	UK	Possible IPA	AML	Cardiff I
Ca15	F	67	UK	Possible IPA	AML	Cardiff I
Ca16	M	63	UK	Possible IPA	AML	Cardiff I
Ca17	M	48	UK	Possible IPA	AML/MDS	Cardiff I
P23	M	52	Germany	Possible IPA	MDS	Würzburg
P33	M	68	Germany	Possible IPA	PCL	Würzburg
P85	M	48	Germany	Possible IPA	AML	Würzburg
P31	M	68	Germany	Control	AML	Würzburg
P44	M	77	Germany	Control	MDS	Würzburg
P53	M	59	Germany	Control	AML	Würzburg

Supplementary Table S2: Clinical information of COVID-19 patients included in independent patient cohort (Cardiff II). Patients samples were collected at Public Health Wales Microbiology Cardiff (Cardiff, United Kingdom).

Patient ID	Sex	Age	Origin	Status	Underlying disease
**C1**	M	58	UK	CAPA	COVID-19
**C2**	M	53	UK	CAPA	COVID-19
**C3**	M	83	UK	CAPA	COVID-19
**C4**	F	53	UK	CAPA	COVID-19
**C5**	M	75	UK	CAPA	COVID-19
**C6**	M	63	UK	control	COVID-19
**C7**	F	59	UK	control	COVID-19
**C8**	M	71	UK	control	COVID-19
**C9**	M	64	UK	control	COVID-19
**C10**	F	45	UK	control	COVID-19
**C11**	M	56	UK	control	COVID-19
**C12**	F	45	UK	control	COVID-19
**C13**	M	63	UK	control	COVID-19
**C14**	F	46	UK	control	COVID-19
**C15**	M	58	UK	control	COVID-19
**C16**	M	53	UK	control	COVID-19
**C17**	F	55	UK	control	COVID-19
**C18**	M	36	UK	control	COVID-19
**C19**	M	59	UK	control	COVID-19
**C20**	M	63	UK	control	COVID-19
**C21**	M	74	UK	control	COVID-19
**C22**	F	62	UK	control	COVID-19
**C23**	F	62	UK	control	COVID-19
**C24**	M	48	UK	control	COVID-19
**C25**	M	58	UK	control	COVID-19
**C26**	M	49	UK	control	COVID-19
**C27**	M	81	UK	control	COVID-19
**C28**	M	38	UK	control	COVID-19
**C29**	F	52	UK	control	COVID-19
**C30**	M	47	UK	control	COVID-19
**C31**	M	70	UK	control	COVID-19
**C32**	M	50	UK	control	COVID-19
**C33**	M	51	UK	control	COVID-19
**C34**	M	57	UK	control	COVID-19
**C35**	M	69	UK	control	COVID-19
**C36**	M	73	UK	control	COVID-19
**C38**	F	58	UK	control	COVID-19
**C39**	M	70	UK	control	COVID-19
**C40**	M	61	UK	control	COVID-19
**C41**	M	54	UK	control	COVID-19
**C42**	M	68	UK	control	COVID-19
**C43**	F	50	UK	control	COVID-19
**C44**	M	57	UK	control	COVID-19
**C45**	M	47	UK	control	COVID-19
**C46**	M	40	UK	control	COVID-19
**C47**	M	45	UK	control	COVID-19
**C48**	M	83	UK	control	COVID-19
**C49**	F	71	UK	control	COVID-19
**C50**	M	56	UK	control	COVID-19
**C51**	M	68	UK	control	COVID-19
**C52**	M	58	UK	CAPA	COVID-19
**C53**	M	74	UK	CAPA	COVID-19
**C54**	F	73	UK	CAPA	COVID-19
**C55**	F	53	UK	CAPA	COVID-19
**C56**	M	74	UK	CAPA	COVID-19
**C57**	F	43	UK	CAPA	COVID-19
**C58**	M	60	UK	CAPA	COVID-19
**C59**	F	69	UK	CAPA	COVID-19
**C60**	M	32	UK	CAPA	COVID-19
**C61**	M	85	UK	CAPA	COVID-19
**C62**	F	61	UK	CAPA	COVID-19
**C63**	F	74	UK	CAPA	COVID-19
**C64**	F	55	UK	CAPA	COVID-19
**C65**	M	59	UK	CAPA	COVID-19
**C66**	M	61	UK	CAPA	COVID-19

Supplementary Table S3: Summary of gene abundance estimation performed using featureCounts (R package Rsubread version 1.34.0).

Sample	Raw Reads	% GC	% rRNA	% GC	Quality reads in Million	% Low Quality	Reads Mapped Uniquely	% of Reads Uniquely Aligned	% Uniquely Assigned to genes	Reads Assigned to genes	Genome Coverage	Exon Coverage
P043-1	9358502	47%	4.3%	46%	8857854	1.1%	8523732	96,23%	50.5%	4,7	0,20	2,39
P043-4	9481139	47%	1.4%	46%	9247172	1.1%	8932617	96,60%	49.3%	4,7	0,20	2,43
P043-5	9780406	48%	2.3%	47%	9456341	1.0%	9125202	96,50%	48.0%	4,8	0,21	2,43
P043-6	9311518	49%	3.6%	49%	8875215	1.1%	8552523	96,36%	50.9%	4,8	0,20	2,50
P043-7	9416538	50%	4.1%	49%	8923383	1.1%	8607040	96,45%	52.3%	5	0,20	2,60
P043-8	11881196	50%	3.8%	50%	11313420	1.0%	10960319	96,88%	49.1%	6,2	0,25	3,25
P043-9	9651508	48%	2.5%	47%	9302860	1.1%	8988839	96,62%	50.4%	5	0,21	2,55
P043-10	9477191	48%	1.8%	47%	9208075	1.1%	8899176	96,65%	56.6%	5,4	0,20	2,81
P043-11	8739514	51%	34.1%	47%	5693697	1.1%	5494951	96,51%	55.0%	3,3	0,13	1,72
P043-12	9076655	48%	3.7%	47%	8643989	1.1%	8357921	96,69%	53.7%	4,9	0,19	2,52
P043-13	8699439	49%	3.0%	48%	8345986	1.1%	8062384	96,60%	53.8%	4,7	0,18	2,46
P043-14	9588937	47%	2.9%	47%	9216169	1.0%	8912999	96,71%	49.6%	4,7	0,20	2,43
P043-15	9978357	47%	3.6%	47%	9514198	1.1%	9190034	96,59%	51.0%	5	0,21	2,57
P043-16	8884480	49%	2.9%	48%	8532677	1.1%	8256032	96,76%	55.2%	5	0,19	2,59
P043-17	9650267	47%	2.9%	46%	9277916	1.0%	8973363	96,72%	52.5%	5,1	0,21	2,61
P043-18	9034000	48%	2.9%	48%	8678646	1.1%	8390165	96,68%	57.5%	5,2	0,19	2,70
P043-19	10021613	47%	3.2%	47%	9595325	1.1%	9284230	96,76%	52.6%	5,3	0,21	2,71
P043-20	10520435	48%	2.9%	48%	10101343	1.1%	9740441	96,43%	58.2%	6,2	0,22	3,22
P018-1	7765812	52%	12.4%	49%	6715798	1.3%	6254038	93,12%	50.8%	3,6	0,14	1,87
P018-5	9324119	47%	1.5%	47%	9087424	1.0%	8770431	96,51%	52.7%	4,9	0,20	2,54
P018-7	10492827	46%	1.2%	46%	10256436	1.1%	9917960	96,70%	46.8%	4,9	0,23	2,48
P018-12	8781298	49%	4.2%	49%	8311834	1.1%	8021853	96,51%	48.7%	4,3	0,18	2,20
P018-13	9263021	49%	6.0%	48%	8603587	1.2%	8296450	96,43%	44.6%	4,1	0,19	2,07
P018-14	10010778	50%	6.3%	49%	9268113	1.2%	8924350	96,29%	44.3%	4,4	0,20	2,22
P018-15	9814688	47%	2.9%	46%	9426602	1.1%	9107046	96,61%	45.5%	4,4	0,21	2,24
P018-16	8081534	47%	3.4%	47%	7718036	1.1%	7458731	96,64%	53.7%	4,3	0,17	2,20
P018-17	10076309	46%	2.7%	46%	9694836	1.1%	9381022	96,76%	46.1%	4,6	0,22	2,34
P018-18	8981551	48%	5.5%	48%	8397992	1.1%	8121547	96,71%	47.6%	4,2	0,19	2,17
P018-19	7742268	49%	8.3%	48%	7019885	1.1%	6778483	96,56%	45.0%	3,4	0,16	1,71
P055-3	10356812	46%	1.3%	46%	10118839	1.0%	9765799	96,51%	58.3%	6,1	0,22	3,21
P055-4	12510315	45%	1.0%	45%	12256512	1.0%	11883231	96,95%	55.2%	7	0,27	3,58
P055-5	11829607	45%	1.7%	45%	11513443	1.0%	11155978	96,90%	53.0%	6,3	0,26	3,25
P055-6	12092441	47%	3.8%	46%	11502769	1.1%	11141080	96,86%	46.7%	5,7	0,26	2,94
P055-7	8777266	47%	5.2%	46%	8240300	1.0%	7972780	96,75%	46.2%	4,1	0,18	2,09
P055-8	12877847	50%	4.0%	49%	12244264	0.9%	11836258	96,67%	46.2%	6,1	0,27	3,15
P055-9	11292252	46%	3.7%	46%	10765410	1.0%	10414124	96,74%	47.9%	5,4	0,24	2,73
P055-10	12006351	48%	6.8%	47%	11059279	1.2%	10661254	96,40%	41.6%	4,9	0,24	2,42
P055-11	12401680	47%	5.6%	46%	11582401	1.1%	11175661	96,49%	43.9%	5,3	0,26	2,64
P063-4	12031263	45%	1.2%	45%	11763369	1.0%	11362287	96,59%	50.0%	6	0,26	3,09
P063-5	14263625	45%	0.9%	45%	13995051	1.0%	13531764	96,69%	47.9%	6,8	0,31	3,48
P063-7	10936707	46%	1.0%	45%	10712583	1.0%	10368167	96,78%	56.1%	6,2	0,24	3,16
P063-9	11483840	46%	1.6%	45%	11187334	1.0%	10817231	96,69%	53.9%	6,2	0,25	3,18
P063-10	11449341	46%	3.3%	45%	10964279	1.0%	10614890	96,81%	48.7%	5,6	0,24	2,84
P063-11	11692711	48%	4.2%	47%	11084640	1.1%	10708832	96,61%	52.1%	6,1	0,25	3,09
P015-12	8980426	48%	6.3%	47%	8315986	1.2%	8012495	96,35%	45.5%	4,1	0,18	2,04
P015-13	11486324	48%	7.4%	47%	10509939	1.2%	10124783	96,34%	46.2%	5,2	0,23	2,63
P015-14	11071549	51%	5.7%	50%	10340728	0.9%	9962277	96,34%	42.5%	4,9	0,23	2,49
P015-15	9641178	49%	7.2%	48%	8849782	1.1%	8560987	96,74%	44.1%	4,3	0,20	2,15
P015-16	10454145	48%	6.9%	47%	9629069	1.1%	9300967	96,59%	43.0%	4,5	0,21	2,24
P015-17	10108313	47%	6.0%	46%	9399313	1.1%	9075278	96,55%	44.5%	4,5	0,21	2,26
P015-18	10599693	47%	5.8%	46%	9879035	1.1%	9519885	96,36%	44.5%	4,7	0,22	2,31
P015-19	11577943	46%	4.5%	45%	10940728	1.0%	10575296	96,66%	50.0%	5,7	0,24	2,90
P015-20	10345331	46%	3.2%	45%	9908831	1.0%	9567651	96,56%	53.4%	5,5	0,22	2,80
P015-21	11603638	45%	2.4%	44%	11202089	1.0%	10830222	96,68%	48.8%	5,6	0,25	2,77
P053-10	10840409	50%	9.2%	48%	9724356	1.2%	9342179	96,07%	48.1%	5,1	0,21	2,54
P053-12	11142686	48%	6.9%	47%	10258247	1.1%	9873151	96,25%	48.2%	5,3	0,23	2,67
P053-13	10742358	48%	4.6%	47%	10143706	1.0%	9795513	96,57%	51.1%	5,7	0,22	2,88
P053-14	9656232	47%	3.9%	46%	9173179	1.1%	8856613	96,55%	45.4%	4,4	0,20	2,21
P053-16	8771870	47%	4.9%	46%	8247200	1.1%	7965873	96,59%	48.7%	4,2	0,18	2,13
P053-17	9405021	46%	5.6%	45%	8779592	1.1%	8481445	96,60%	49.8%	4,6	0,19	2,33
P053-18	9247203	46%	4.0%	46%	8778129	1.1%	8484953	96,66%	44.9%	4,2	0,19	2,06
P053-19	9271655	48%	6.2%	47%	8601920	1.1%	8289437	96,37%	49.2%	4,6	0,19	2,32
P018-6	9858295	46%	1.3%	45%	9614675	1.1%	9302775	96,76%	48.4%	4,8	0,21	2,42
P063-8	9286052	48%	2.3%	48%	8964705	1.1%	8637910	96,35%	61.7%	5,7	0,20	2,98
P053-15	8796929	51%	5.6%	50%	8202955	1.2%	7882557	96,09%	53.2%	4,8	0,18	2,46
P015-22	8977998	52%	6.8%	51%	8248551	1.4%	7897106	95,74%	39.0%	3,7	0,18	1,81

Supplementary Table S4: Summary of the samples used for the balanced data analysis (first five weeks after IPA onset). Patients samples were were collected at the University Hospital of Würzburg (Würzburg, Germany).

Sample	After alloSCT (days)	RelTime (days)
P015-15	82	0
P015-16	92	10
P015-17	106	24
P015-18	113	31
P053-13	82	0
P053-15	91	9
P053-16	100	18
P053-17	113	31
P055-4	12	4
P055-5	15	7
P055-8	28	20
P055-9	41	33
P063-4	12	4
P063-5	15	7
P063-9	28	20
P063-10	40	32
P043-7	46	4
P043-9	53	11
P043-11	60	18
P043-13	67	25
P018-13	46	4
P018-14	56	14
P018-16	67	25
P018-17	70	28

Supplementary Table S7: Mean serum protein levels of Caspase-3 and IL-8, investigated in female and male probable IPA cases (IPA) and controls (CTRL) collected at Public Health Wales Microbiology Cardiff (Cardiff, United Kingdom).

**Analyte**	**Females**		**Males**	
**Mean (pg/ml)**	**CTRL**	**IPA**	**CTRL**	**IPA**
**Caspase -3**	388,8	1457	81,92	2066
**IL-8**	7,668	53,75	8,634	146,3

Supplementary Figure S1: Matched case-control log2FC for MMP9 and ITGB3 of profiled alloSCT patients (Würzburg) in all investigated time-points.

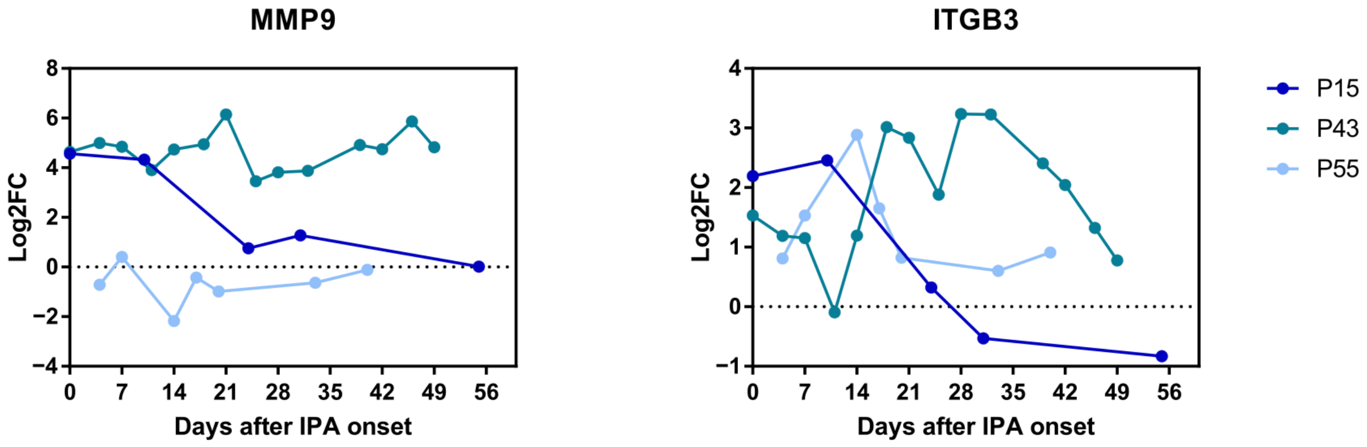

Supplementary Figure S2: Serum levels of MMP9, VEGFA, galectin-2, serpine-1 and ITGB3 in probable IPA cases and their matched controls. **(a)** across all investigated patients samples (Würzburg, Germany). **(b)** within first 5 weeks after IPA onset (Würzburg, Germany; balanced dataset). **(c)** in patient sera obtained from Cardiff, United Kingdom (Cardiff I). Significant differences between cases and controls were investigated by Mann-Whitney test (* $p<0.05$, ** $p<0.01$, *** $p<0.001$, **** $p<0.0001$).

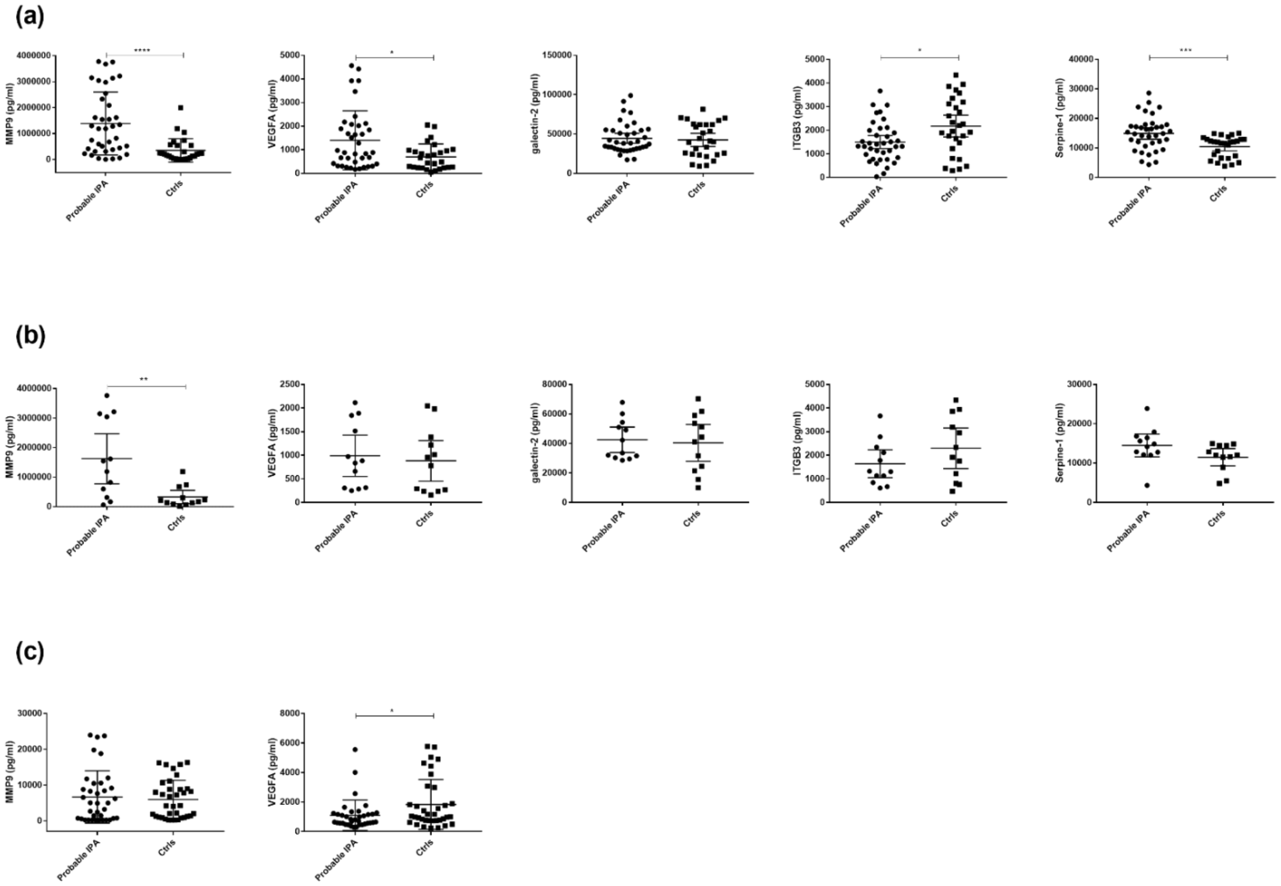

Supplement: Supplementary file 1 [file jof-08-00171-s001.zip › Supplementary_material.pdf]
